# Diabetes Mellitus as a Risk Factor for Severe Disease and Mortality Among Patients with Melioidosis: A Systematic Review and Meta-Analysis

**DOI:** 10.3390/life16020361

**Published:** 2026-02-21

**Authors:** Jongkonnee Thanasai, Anchalee Chittamma, Supphachoke Khemla, Atthaphong Phongphithakchai, Moragot Chatatikun, Jitbanjong Tangpong, Sa-ngob Laklaeng, Wiyada Kwanhian Klangbud

**Affiliations:** 1 Faculty of Medicine, Mahasarakham University, Mahasarakham 44000, Thailand; jongkonnee@msu.ac.th; 2 Department of Pathology, Faculty of Medicine Ramathibodi Hospital, Mahidol University, Bangkok 10400, Thailand; 3 Division of Infectious Diseases, Department of Internal Medicine, Nakhon Phanom Hospital, Nakhon Phanom 48000, Thailand; sup.mednkp@gmail.com; 4 Nephrology Unit, Division of Internal Medicine, Faculty of Medicine, Prince of Songkla University, Songkhla 90110, Thailand; 5 School of Allied Health Sciences, Walailak University, Nakhon Si Thammarat 80160, Thailand; 6 Research Excellence Center for Innovation and Health Products (RECIHP), Walailak University, Nakhon Si Thammarat 80160, Thailand; 7 Medical Technology Program, Faculty of Science, Nakhon Phanom University, Nakhon Phanom 48000, Thailand; 8 Faculty of Medicine, Nakhon Phanom University, Nakhon Phanom 48000, Thailand

**Keywords:** *Burkholderia pseudomallei*, diabetes mellitus, disease severity, melioidosis, mortality

## Abstract

**Background**: Melioidosis is a potentially fatal infectious disease caused by *Burkholderia pseudomallei* and is highly prevalent in tropical regions. Diabetes mellitus (DM) is the most common comorbidity among patients with melioidosis and is a well-established risk factor for acquiring the infection. However, the impact of diabetes on disease severity and mortality remains uncertain. **Methods**: We conducted a systematic review and meta-analysis of observational studies to evaluate the association between diabetes mellitus and severe clinical outcomes in patients with melioidosis. PubMed, Embase, and Scopus were searched from database inception to 6 January 2026. Outcomes of interest included bacteremia, septic shock, intensive care unit (ICU) admission, and mortality. Pooled odds ratios with 95% confidence intervals (CIs) were calculated using random-effects models. Heterogeneity was assessed using the *I*^2^ statistic. The review was registered in PROSPERO (CRD420251237028). **Results**: Twelve studies comprising patients from Southeast Asia, Australia, and South Asia were included. Diabetes prevalence among patients with melioidosis ranged from 31% to 76%. Meta-analysis showed no significant association between diabetes mellitus and bacteremia (OR 1.48, 95% CI 0.97–2.27), ICU admission (OR 1.31, 95% CI 0.43–3.99), septic shock (OR 0.67, 95% CI 0.39–1.16), or mortality (OR 0.82, 95% CI 0.66–1.03). Subgroup analysis revealed lower mortality among patients with diabetes in Southeast Asia (OR 0.74, 95% CI 0.61–0.91), while no significant association was observed in Australia. Heterogeneity varied across outcomes and regions. **Conclusions**: Although diabetes mellitus is a major risk factor for acquiring melioidosis, our findings suggest that it is not consistently associated with increased disease severity or mortality once infection occurs. These results should be interpreted cautiously given the limited number of included studies, heterogeneity across outcomes, and potential residual confounding. Further prospective studies are required to better define the underlying biological and healthcare-related mechanisms.

## 1. Introduction

Melioidosis is a severe and often fatal infectious disease caused by *Burkholderia pseudomallei*, a Gram-negative bacterium endemic to tropical and subtropical regions, particularly Southeast Asia, northern Australia, and South Asia. The pathogen, commonly found in soil and surface water, infects humans through percutaneous inoculation, inhalation, or ingestion, leading to a wide spectrum of clinical manifestations that range from localized abscesses to fulminant septicemia and multi-organ failure [1,2]. Despite advances in microbiological diagnostics and antimicrobial therapy, the disease remains associated with high mortality—below 10% in well-resourced settings but exceeding 40% in parts of Southeast Asia and South Asia [3]. Among the predisposing factors, diabetes mellitus (DM) consistently emerges as the most significant and prevalent risk factor. Epidemiological studies have shown that up to 60–80% of melioidosis patients have diabetes, underscoring its profound contribution to disease susceptibility and severity [4,5]. Diabetes mellitus is consistently reported as the most common comorbidity among patients with melioidosis and a well-established risk factor for acquiring infection; however, its relationship with disease severity and mortality appears heterogeneous across studies and regions [6,7]. Given the escalating global prevalence of diabetes—particularly in low- and middle-income countries where melioidosis is endemic—the convergence of these two diseases poses a growing threat to public health and healthcare systems [8].

Although the relationship between diabetes and melioidosis has been widely reported, the literature remains inconsistent and fragmented. For instance, Hanson et al. (2021) demonstrated that diabetes and socioeconomic disadvantage independently increased mortality in Australian patients [5], whereas Zueter et al. (2016) identified diabetes as an independent predictor of death (odds ratio 3.0) in Malaysian patients [3], with septic shock and advanced age further compounding the risk. In contrast, Zheng et al. (2023) found a high prevalence of diabetes among melioidosis cases in China but failed to demonstrate a consistent relationship between glycemic status and mortality [4]. These discrepancies likely stem from regional variations in healthcare access, diagnostic timing, and clinical management [9,10]. Furthermore, many earlier studies were limited by small sample sizes, retrospective designs, inconsistent definitions of clinical outcomes, and inadequate adjustment for confounders such as age, sex, and comorbidities [11]. To date, no comprehensive meta-analysis has quantitatively synthesized the evidence to clarify whether diabetes independently influences the risk of severe clinical outcomes—including bacteremia, septic shock, ICU admission, and mortality—across diverse endemic settings.

To address these gaps, the present study conducts a systematic review and meta-analysis to quantitatively evaluate the association between diabetes mellitus and severe clinical outcomes among patients with melioidosis. Specifically, it aims to determine whether diabetes independently predicts poor outcomes while accounting for regional and methodological heterogeneity. By integrating evidence from multiple observational cohorts, this research provides the first robust pooled estimates of diabetes-associated risk, offering insights essential for clinical risk stratification, early management, and resource allocation in endemic regions. Ultimately, the findings are expected to contribute to more targeted clinical interventions and inform public health strategies aimed at mitigating the combined burden of diabetes and melioidosis in vulnerable populations.

## 2. Materials and Methods

### 2.1. Study Design

This systematic review and meta-analysis aimed to assess the impact of diabetes mellitus (DM) on the severity of melioidosis and its association with mortality. The study was conducted in accordance with the Preferred Reporting Items for Systematic Reviews and Meta-Analyses (PRISMA) guidelines [12] and was prospectively registered in the PROSPERO database (registration number: CRD420251237028). A comprehensive search was conducted across PubMed, Scopus, and Embase from database inception to 6 January 2026.

### 2.2. Search Strategies

A comprehensive and systematic literature search was conducted to identify all relevant studies evaluating diabetes mellitus as a risk factor for severe disease and mortality among patients with melioidosis. The search was performed independently in three electronic databases—PubMed, Embase, and Scopus—from database inception to 6 January 2026. Search strategies were developed using a combination of controlled vocabulary terms (Medical Subject Headings [MeSH] in PubMed and Emtree terms in Embase) and free-text keywords related to both diabetes mellitus and melioidosis, as shown in Supplementary Table S1.

For diabetes mellitus, the search included terms such as “diabetes mellitus,” “diabetes,” “diabetic,” “DM,” and truncations (e.g., diabet*). For melioidosis, terms included “melioidosis,” “*Burkholderia pseudomallei* infection,” “Whitmore’s disease,” and related synonyms. Boolean operators (AND, OR) were applied to combine concepts appropriately, and database-specific syntax was used to optimize sensitivity. No restrictions were applied regarding language, publication status, or study design at the search stage to ensure comprehensive retrieval.

During the first phase of screening, titles and abstracts were independently reviewed by two reviewers to exclude records that were clearly irrelevant. Records were excluded at this stage if they did not involve melioidosis, were not conducted in human populations, were non-original research (e.g., reviews, editorials, conference abstracts), were case reports or case series, or did not assess diabetes mellitus as an exposure of interest. Full-text articles were retrieved for records that could not be confidently excluded based on title and abstract alone.

### 2.3. Inclusion and Exclusion Criteria

Studies were eligible for inclusion if they were observational in design, involved culture-confirmed melioidosis, and evaluated the association between diabetes mellitus and clinical outcomes, including bacteremia, septic shock, intensive care unit (ICU) admission, or mortality. Eligible study designs included prospective cohort studies, retrospective cohort studies, case–control studies, and cross-sectional studies that reported comparative data by diabetes status.

Studies were excluded if they were case reports or case series, did not include diabetes mellitus as an exposure of interest, lacked a comparator group, or did not report extractable outcome data relevant to the review objectives. Study selection was performed sequentially, with records excluded during title and abstract screening followed by full-text review, according to these predefined inclusion and exclusion criteria, as outlined in the PRISMA flow diagram.

### 2.4. Data Extraction

Data were extracted independently by two reviewers (Jo.T. and W.K.K.) using a standardized form. Discrepancies were resolved by discussion, and if disagreement persisted, a third reviewer (M.C.) adjudicated. Extracted items included: (i) study characteristics (year, country, setting, design, inclusion criteria, sample size); (ii) patient characteristics (age, sex); (iii) diabetes ascertainment method (history, medication use, laboratory definition as reported); (iv) other comorbidities reported (e.g., chronic kidney disease, hazardous alcohol use, chronic lung disease); and (v) outcomes (bacteremia, septic shock, ICU admission, mortality) and their definitions/timeframes as reported. The mortality outcome was extracted according to each study’s reporting (in-hospital, 28-day, or 90-day). When multiple timepoints were reported, we prioritized the most commonly reported endpoint across studies (pre-specified hierarchy: in-hospital > 28-day > 90-day), and we performed sensitivity analysis where feasible. Septic shock definitions were accepted as reported by the original studies; where explicit criteria were provided (e.g., blood pressure/vasopressor requirements, lactate), these were recorded.

### 2.5. Quality Assessment

Study quality was independently assessed by two reviewers (Jo.T. and W.K.K) using the Newcastle–Ottawa Scale (NOS) [13]. Discrepancies were resolved by discussion, and if disagreement persisted, a third reviewer (M.C.) adjudicated. The scale evaluates selection, comparability, and outcome/exposure domains for observational studies. Disagreements were resolved by consensus.

Studies were scored out of nine stars and classified as high (7–9), moderate (4–6), or low quality (≤3). Most studies were of moderate to high quality. Common limitations included retrospective design and variable outcome definitions. Quality scores were considered in sensitivity analyses, and no study was excluded based on quality alone.

### 2.6. Publication Bias Assessment

Publication bias was assessed by visual inspection of funnel plots. However, given the limited number of studies included in each analysis, the assessment of publication bias is inherently underpowered, and funnel plot asymmetry cannot be reliably interpreted. Accordingly, any apparent asymmetry should be considered exploratory, and the presence or absence of publication bias cannot be conclusively determined [14].

### 2.7. Statistical Analysis:

Meta-analyses were conducted using a random-effects model to account for between-study variability. Pooled odds ratios (ORs) were derived from crude (unadjusted) estimates, calculated from 2 × 2 contingency tables where raw data were available. Adjusted effect estimates were not pooled because adjustment strategies and covariates varied substantially across studies, limiting comparability. This limitation is addressed in the Discussion.

Not all included studies directly reported ORs. When ORs were not explicitly provided, they were calculated from extractable raw data. Studies reporting relative risks or other effect measures without sufficient raw data were not transformed or pooled, and no conversion from relative risk to odds ratio was performed.

Statistical heterogeneity was assessed using the *I*^2^ statistic and interpreted using conventional thresholds: low heterogeneity (*I*^2^ < 25%), moderate heterogeneity (25–50%), and substantial heterogeneity (>50%). Statistical analyses were performed using R software (version 3.6.1), with two-sided *p* values < 0.05 considered statistically significant.

## 3. Results

### 3.1. Study Selection

**Figure 1:**
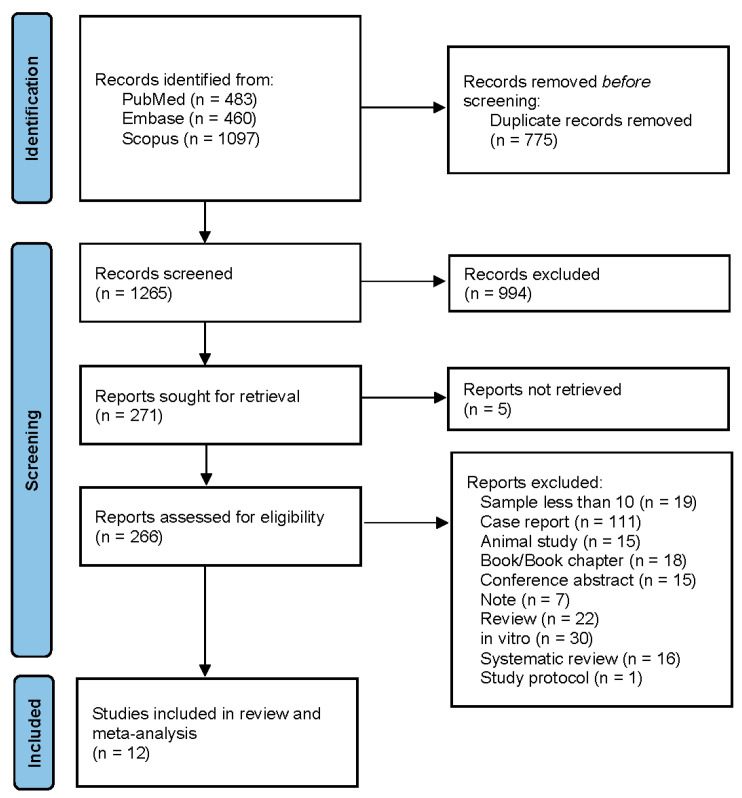
PRISMA flow diagram of study selection.

The systematic search identified a total of 2040 records across PubMed (483), Embase (460), and Scopus (1,097) databases. After removal of duplicate records (*n* = 775), 1265 records were screened by title and abstract. Of these, 994 records were excluded. Full texts were sought for 271 reports, of which five reports could not be retrieved. A total of 266 full-text articles were assessed for eligibility. Of these, 254 articles were excluded for the following reasons: sample size less than 10 (*n* = 19), case report (*n* = 111), animal study (*n* = 15), book/book chapter (*n* = 18), conference abstract (n = 15), note (*n* = 7), review article (*n* = 22), in vitro study (*n* = 30), systematic review (*n* = 16), and study protocol (*n* = 1). Finally, 12 studies met the inclusion criteria and were included in the systematic review and meta-analysis. The details are in Figure 1.

### 3.2. Characteristics of Included Studies

The 12 included studies encompassed a wide geographic distribution across melioidosis-endemic regions, including Thailand (*n* = 5), Australia (*n* = 4), India (*n* = 1), Malaysia (*n* = 1), and Sri Lanka (*n* = 1). Study designs were heterogeneous and included prospective cohort studies, retrospective cohort analyses, a longitudinal study, a case–control study, and a nationwide case-finding study. Sample sizes varied considerably, ranging from small hospital-based cohorts of 32 patients to large multicenter prospective studies involving up to 1160 participants.

Diabetes mellitus was consistently reported as the most common underlying comorbidity across all included studies. The prevalence of diabetes among patients with melioidosis ranged from approximately 31–39% in several Thai and Australian cohorts, to markedly higher proportions in South and Southeast Asia, reaching 71.7% in Malaysia and 76.2% in India. The majority of studies evaluated severe clinical outcomes, primarily mortality, reported as in-hospital, 28-day, or 90-day case-fatality rates. Additional outcomes included bacteremia, septic shock, ICU admission, pneumonia, and acute kidney injury.

Marked regional differences in case-fatality rates were observed. Studies from Australia have demonstrated a progressive decline in mortality over time, with reported rates as low as 9%. In contrast, mortality remained substantially higher in Southeast Asia and South Asia, ranging from 26% to 52.2%. Detailed characteristics of the included studies are presented in Table 1.

### 3.3. Quality of Included Studies

The methodological quality of the included studies, assessed using the Newcastle–Ottawa Scale (NOS), is summarized in Supplementary Table S2. Overall, study quality was moderate to high, with total scores ranging from 7 to 8 stars. Most studies demonstrated good representativeness of study populations and reliable ascertainment of diabetes mellitus and clinical outcomes. Comparability was generally adequate, with the majority of studies adjusting for key confounders such as age and sex. Common limitations included retrospective study design, incomplete adjustment for additional comorbidities, and heterogeneity in outcome definitions, particularly for ICU admission and septic shock. No study was judged to be at high risk of bias, and all were retained for quantitative synthesis.

### 3.4. Publication Bias

Funnel plots did not demonstrate clear visual asymmetry; however, given the small number of studies included for each outcome, the assessment of publication bias is underpowered and cannot be interpreted definitively (Supplementary Figure S1). The plots appeared relatively symmetric, indicating that the results of this meta-analysis are unlikely to be significantly influenced by publication bias.

However, as the number of studies included in the analysis was small, the ability to conclusively assess publication bias is limited. Thus, while the visual inspection suggests minimal publication bias, caution is still warranted due to the small number of studies included for each outcome.

### 3.5. Quantitative Synthesis

#### 3.5.1. Bacteremia

Six studies contributed data for the analysis of bacteremia [8,14,15,19,22,24]. In the pooled random-effects meta-analysis, diabetes mellitus was not statistically significantly associated with bacteremia; however, the pooled estimate suggested a trend toward increased odds, with wide confidence intervals indicating limited precision (pooled OR 1.48, 95% CI 0.97–2.27). Although the point estimate suggested a trend toward increased odds of bacteremia in patients with diabetes, the confidence interval crossed unity, indicating statistical non-significance.

**Figure 2:**
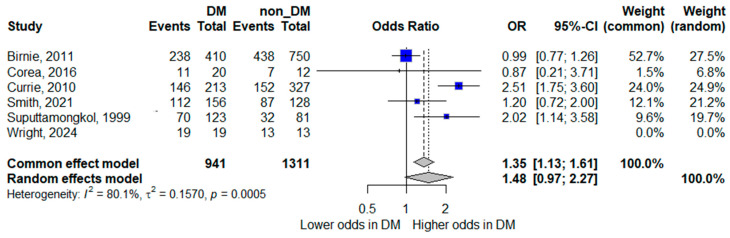
Forest plot of the association between diabetes mellitus and bacteremia. Squares represent study-specific odds ratios (ORs) proportional to study weight; horizontal lines indicate 95% confidence intervals (CIs). The vertical line marks no effect (OR = 1). The diamond represents the pooled OR with its 95% CI. The included studies correspond to references [8,15,16,20,23,25].

Substantial heterogeneity was observed (*I*^2^ = 80.1%), reflecting marked between-study variability. This heterogeneity likely reflects differences in methodological and clinical factors, including variation in blood culture sampling practices (e.g., number and timing of cultures, blood volume collected, and laboratory methods or automation), differences in case definitions and inclusion criteria, and heterogeneity in healthcare settings and diagnostic pathways across regions. The forest plot is shown in Figure 2.

#### 3.5.2. ICU Admission

Three studies reported ICU admission as an outcome [15,18,20]. The pooled random-effects analysis did not demonstrate a statistically significant association between diabetes mellitus and ICU admission; however, the pooled estimate suggested a trend toward increased odds, with wide confidence intervals indicating limited precision (pooled OR 1.31, 95% CI 0.43–3.99). The wide confidence interval indicates considerable imprecision, largely attributable to the small number of contributing studies and events.

**Figure 3:**
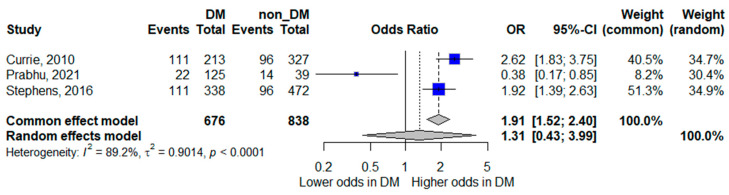
Forest plot of the association between diabetes mellitus and ICU admission. Squares represent study-specific odds ratios (ORs) proportional to study weight; horizontal lines indicate 95% confidence intervals (CIs). The vertical line marks no effect (OR = 1). The diamond represents the pooled OR with its 95% CI. The included studies correspond to references [16,19,21].

Heterogeneity was very high (*I*^2^ = 89.2%), highlighting substantial variation across study settings and healthcare systems. These findings suggest that the relationship between diabetes and ICU admission may be context-dependent and influenced by local admission criteria and resource availability. The forest plot is presented in Figure 3.

#### 3.5.3. Septic Shock

Two studies assessed septic shock as an outcome [8,18]. The pooled random-effects model showed diabetes mellitus was not statistically significantly associated with septic shock, although the pooled estimate suggested a trend toward lower odds of septic shock; the confidence interval crossed unity, indicating inconclusive evidence (pooled OR 0.67, 95% CI 0.39–1.16). While the point estimate suggested lower odds of septic shock among patients with diabetes, the confidence interval included the null value.

**Figure 4:**
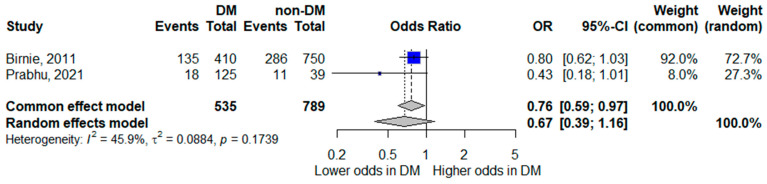
Forest plot of the association between diabetes mellitus and septic shock. Squares represent study-specific odds ratios (ORs) proportional to study weight; horizontal lines indicate 95% confidence intervals (CIs). The vertical line marks no effect (OR = 1). The diamond represents the pooled OR with its 95% CI. The included studies correspond to references [8,19].

Moderate heterogeneity was observed (*I*^2^ = 45.9%). Given the limited number of studies and differences in definitions and diagnostic thresholds for septic shock, these results should be interpreted cautiously. The corresponding forest plot is shown in Figure 4.

#### 3.5.4. Mortality

Seven studies reported mortality outcomes [8,14,15,16,17,21,23] and were included in the quantitative synthesis. The pooled random-effects meta-analysis showed that diabetes mellitus was not statistically significantly associated with mortality, although the pooled estimate suggested a trend toward lower mortality; the confidence interval crossed unity, indicating inconclusive evidence (pooled OR 0.82, 95% CI 0.66–1.03). Heterogeneity was low (*I*^2^ = 12.5%), indicating relatively consistent findings across studies.

**Figure 5:**
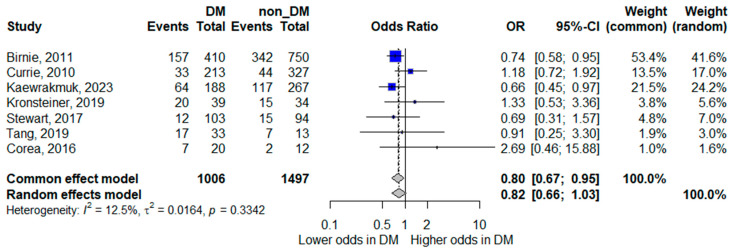
Forest plot of the association between diabetes mellitus and mortality. Squares represent study-specific odds ratios (ORs) proportional to study weight; horizontal lines indicate 95% confidence intervals (CIs). The vertical line marks no effect (OR = 1). The diamond represents the pooled OR with its 95% CI. The included studies correspond to references [8,15,16,17,18,22,24].

Although the pooled point estimate suggested a trend toward lower mortality among patients with diabetes, the confidence interval crossed unity, and therefore no statistically significant difference was demonstrated. The forest plot for mortality is presented in Figure 5.

#### 3.5.5. Subgroup Analyses by Region

Regional subgroup analyses were performed to explore potential differences in outcomes across geographic settings. However, these analyses were based on a limited number of studies, and in some subgroups, estimates were derived from a single study. As such, the results should be interpreted cautiously and considered exploratory rather than definitive. Observed regional patterns may reflect differences in healthcare infrastructure, diagnostic practices, or study design rather than true biological variation, and further studies are required to confirm these findings.

##### Mortality by Region

**Figure 6:**
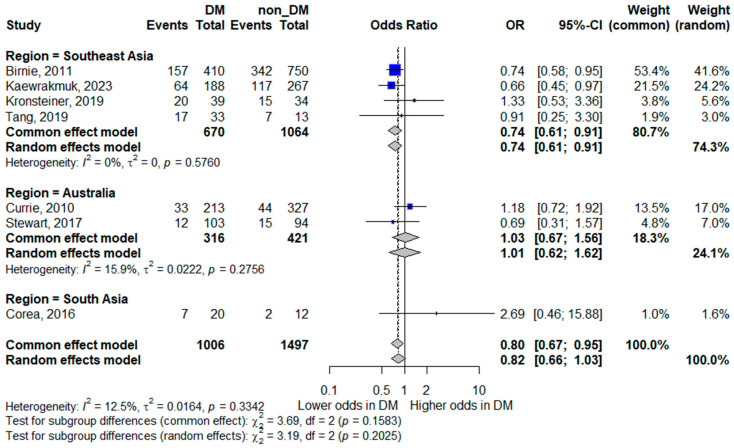
Subgroup analysis of mortality by geographic region. Squares represent study-specific odds ratios (ORs) proportional to study weight; horizontal lines indicate 95% confidence intervals (CIs). The vertical line marks no effect (OR = 1). The diamond represents the pooled OR with its 95% CI. The included studies correspond to references [8,15,16,17,18,22,24].

Regional subgroup analysis revealed notable differences in the association between diabetes and mortality. In Southeast Asia, diabetes mellitus was associated with lower odds of mortality (pooled OR 0.74, 95% CI 0.61–0.91), with no observed heterogeneity (*I*^2^ = 0%), indicating consistent findings across studies in this region. In contrast, studies from Australia showed no significant association between diabetes and mortality (pooled OR 1.01, 95% CI 0.62–1.62), with low heterogeneity (*I*^2^ = 18.2%). South Asia was represented by a single study, yielding a highly imprecise estimate (OR 2.69, 95% CI 0.46–15.88). These findings are illustrated in Figure 6.

##### ICU Admission by Region

**Figure 7:**
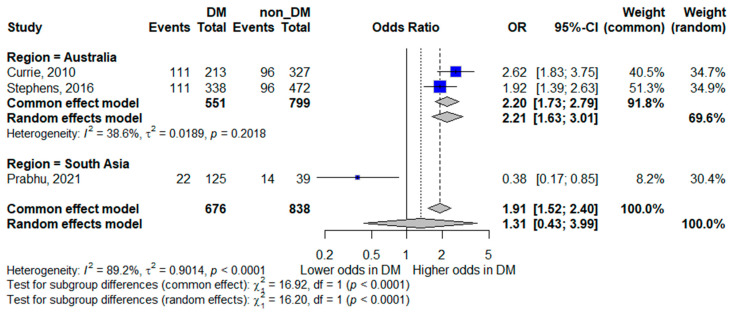
Subgroup analysis of ICU admission by geographic region. Squares represent study-specific odds ratios (ORs) proportional to study weight; horizontal lines indicate 95% confidence intervals (CIs). The vertical line marks no effect (OR = 1). The diamond represents the pooled OR with its 95% CI. The included studies correspond to references [16,19,21].

Regional analysis for ICU admission demonstrated divergent patterns. In Australia, diabetes mellitus was associated with higher odds of ICU admission (pooled OR 2.21, 95% CI 1.63–3.01), with low-to-moderate heterogeneity (*I*^2^ = 38.6%), suggesting a consistent association in this setting. Conversely, the single South Asian study reported lower odds of ICU admission among patients with diabetes (OR 0.38, 95% CI 0.17–0.85). Due to the limited data in this subgroup, these findings should be interpreted with caution. The forest plot is shown in Figure 7.

##### Bacteremia by Region

**Figure 8:**
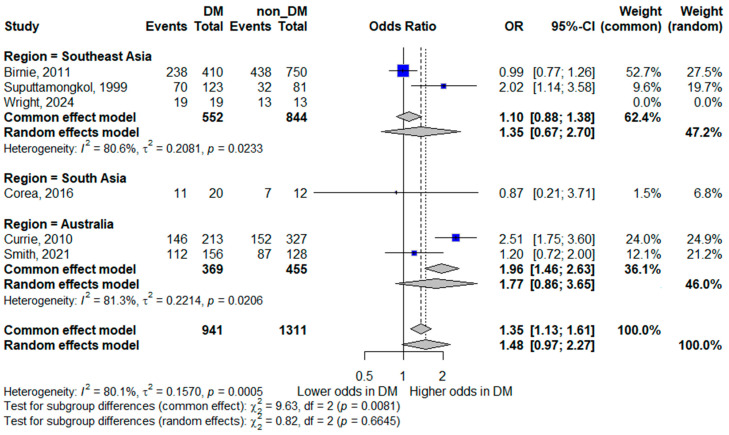
Subgroup analysis of bacteremia by geographic region. Squares represent study-specific odds ratios (ORs) proportional to study weight; horizontal lines indicate 95% confidence intervals (CIs). The vertical line marks no effect (OR = 1). The diamond represents the pooled OR with its 95% CI. The included studies correspond to references [8,15,16,20,23,25].

Subgroup analyses for bacteremia continued to demonstrate substantial heterogeneity within regions. In Southeast Asia, diabetes was not significantly associated with bacteremia (pooled OR 1.35, 95% CI 0.67–2.70; *I*^2^ = 80.6%). Similarly, in Australia, no significant association was observed (pooled OR 1.77, 95% CI 0.86–3.65; *I*^2^ = 81.3%). A single South Asian study reported no significant association (OR 0.87, 95% CI 0.21–3.71). The persistence of high heterogeneity across regions suggests that geographic stratification alone does not fully explain the variability between studies. Methodological differences, patient selection, and clinical management practices likely contribute to these inconsistencies. The forest plot is shown in Figure 8.

## 4. Discussion

This systematic review and meta-analysis evaluated the role of diabetes mellitus (DM) as a risk factor for severe outcomes in melioidosis, an infection caused by *Burkholderia pseudomallei*. Our findings indicate that while diabetes is a major predisposing factor for melioidosis, its role as an independent predictor of adverse clinical outcomes, including bacteremia, septic shock, intensive care unit (ICU) admission, and mortality, is more complex than traditionally assumed.

The consistently high prevalence of diabetes among patients with melioidosis across all included studies aligns with previous reports identifying diabetes as one of the strongest risk factors for acquiring the infection [5,16]. However, our pooled analyses did not demonstrate statistically significant associations between diabetes and disease severity, such as bacteremia, septic shock, or ICU admission. These non-significant findings should be interpreted as inconclusive due to the limited number of studies, wide confidence intervals, and residual heterogeneity.

These results contrast with earlier reports, particularly from Southeast Asia, which described poorer outcomes among patients with diabetes, often in settings with limited access to timely diagnosis and critical care [3,18]. These discrepancies suggest that regional healthcare resources—including diagnostic capacity, antimicrobial availability, and access to intensive care—may substantially influence observed outcomes. In well-resourced settings such as Australia, temporal improvements in diagnostics and clinical management have been associated with lower mortality, whereas older cohorts and studies from resource-limited settings may overestimate severity due to delayed presentation or diagnostic constraints [21,22].

Diabetes is associated with altered innate and adaptive immune function, including dysregulated cytokine production and impaired neutrophil activity [17,26,27]. These alterations could theoretically blunt the magnitude of a cytokine-driven hyperinflammatory response that characterizes severe sepsis and multiorgan dysfunction.

Supportive evidence from immunological studies further suggests that diabetes may modify host immune responses relevant to melioidosis severity. T-cell-mediated immunity, particularly CD4^+^ and CD8^+^ T-cell responses, plays a critical role in controlling *B. pseudomallei* infection. Individuals with diabetes often exhibit impaired T-cell activation and reduced production of key cytokines such as interferon-γ and interleukin-17, which are essential for intracellular pathogen clearance [25]. Oxidative stress associated with chronic hyperglycemia may further impair immune cell function. Importantly, some studies suggest that improved glycemic control can partially restore immune responses, potentially enhancing host defense even among individuals with diabetes [27,28]. However, these immunological mechanisms were not directly assessed in the included studies and should be interpreted as hypothesis-generating.

In addition to biological factors, healthcare-related mechanisms may also contribute to the observed findings. Patients with diabetes often have more frequent healthcare interactions, facilitating earlier diagnosis, prompt blood culture sampling, and timely initiation of antimicrobial therapy. Variations in triage thresholds, clinical monitoring, and admission practices for diabetic patients may further confound the observed relationships between diabetes and disease severity.

Methodological considerations are also important. Residual confounding by age, comorbidities, and healthcare access is likely, as patients without diagnosed diabetes may include those with undiagnosed dysglycemia or other risk factors that independently increase mortality. The non-significant associations observed in this study should therefore be interpreted cautiously, particularly given the wide confidence intervals, limited number of studies, and substantial heterogeneity across outcomes. Furthermore, most studies relied on unadjusted data, and inconsistent covariate adjustments hinder the ability to draw definitive conclusions about causality.

Our analysis also highlights substantial regional heterogeneity in melioidosis outcomes. In well-resourced regions such as northern Australia, early diagnosis and prompt antimicrobial therapy have markedly improved survival, including among patients with diabetes [16,22]. In contrast, in resource-limited settings, delayed diagnosis and restricted access to intensive care continue to contribute to high mortality among diabetic patients, despite the well-established role of diabetes in host susceptibility [18].

This study has several limitations. First, the observational nature of the included studies limits causal inference and increases susceptibility to residual confounding. Second, the pooled estimates were derived from mostly unadjusted data, as adjusted estimates were inconsistently reported and varied across studies, reducing the reliability of the associations. Third, the substantial heterogeneity observed likely reflects differences in study design, diagnostic practices, and healthcare settings. Finally, the lack of data on glycemic control, diabetes duration, and diabetes-related complications, coupled with the small number of studies, reduced statistical power and precluded robust subgroup analyses.

Overall, our findings underscore the need for further research to clarify the immunological and clinical mechanisms linking diabetes and melioidosis outcomes. Future studies should prioritize prospective cohort designs, standardized definitions of disease severity and outcomes, comprehensive adjustment for metabolic and comorbidity profiles, and the incorporation of immune biomarkers, including cytokine responses, T-cell activation, and oxidative stress markers, to better elucidate the complex interactions between diabetes, immune dysfunction, and melioidosis severity.

## 5. Conclusions

In conclusion, diabetes mellitus is strongly associated with susceptibility to melioidosis but does not appear to be consistently associated with increased severity or mortality once infection occurs. However, the available evidence remains inconclusive due to limited statistical power, imprecision, and substantial heterogeneity. These findings should be interpreted cautiously given the limitations, and further prospective studies are required to better define the underlying biological and healthcare-related mechanisms.

## Figures and Tables

**Table 1:** Characteristics of included studies.

Author, Year [Ref]	Study Design	Location	Sample Size (N)	DM (n)	Non-DM (n)	Overall Fatality (%)	Fatality Rate in DM (%)	Main Outcomes Measured
Birnie, 2019 [8]	Prospective cohort	Thailand	1160	410	750	26%	NA	In-hospital mortality; acute kidney injury (AKI); respiratory failure; hypotension.
Corea, 2016 [15]	Case-finding	Sri Lanka	32	20	12	14%	15%	Case fatality rate; clinical spectrum (sepsis, pneumonia, focal infection).
Currie, 2010 [16]	Prospective cohort	Australia	540	213	327	28%	30.0%	Mortality; bacteremia; septic shock; pneumonia; recurrence (relapse/reinfection).
Kronsteiner, 2019 [17]	Longitudinal study	Thailand	135	90	45	34%	NA	28-day mortality; immune correlates of survival; cytokine profiling.
Kaewrakmuk, 2023 [18]	Retrospective study	Thailand	455	188	267	14%	12%	Case fatality rate; bacteremia; pneumonia; average annual incidence.
Prabhu, 2021 [19]	Retrospective cohort	India	164	125	39	43%	NA	AKI; ICU hospitalization; in-hospital mortality.
Smith, 2021 [20]	Retrospective analysis	Australia	284	153	131	26%	51.3%	Disease incidence; case-fatality rate; bacteremia.
Stephens, 2016 [21]	Prospective database	Australia	207	111	96	52.2%	56.7%	Mortality; ICU length of stay; organ support requirements.
Stewart, 2017 [22]	Retrospective series	Australia	197	103	94	15.2%	NA	Case fatality rate; disease recurrence; clinical manifestations.
Suputtamongkol, 1999 [23]	Case–control	Thailand	204	123	81	9–27%	NA	Risk factors for melioidosis and bacteremia; mortality.
Tang, 2019 [24]	Retrospective study	Malaysia	46	33	13	39.8%	34.0%	Mortality rate; ICU admission rate; clinical presentations (pneumonia, abscess).
Wright, 2024 [25]	Prospective cohort	Thailand	32	19	13	31%	NA	28-day mortality; functional and phenotypic cellular immune response changes.

NA indicates that specific mortality for the DM subgroup was not explicitly provided in the study.

## Data Availability

No new data were created.

## Supplementary Materials

The following supporting information can be downloaded at: https://www.mdpi.com/article/10.3390/life16020361/s1, Supplementary Table S1: Search strategy for database querying; Supplementary Table S2: Newcastle–Ottawa Scale (NOS) quality assessment of included studies; Supplementary Figure S1: Funnel plots.

## Abbreviations

The following abbreviations are used in this manuscript:

OR	Odds ratio
CI	Confidence interval
ICU	Intensive care unit
