# Supplementary material for: Diabetes Mellitus as a Risk Factor for Severe Disease and Mortality Among Patients with Melioidosis: A Systematic Review and Meta-Analysis
Source: Life (Basel). 2026 Feb 21;16(2):361. doi: 10.3390/life16020361 (PMC12942006; doi:10.3390/life16020361)
Supplement: Supplementary file 1 [file life-16-00361-s001.zip › Supplementary Figure S1_Funnel plot.pdf]

Diabetes mellitus as a risk factor for severe disease and mortality among patients with melioidosis: a systematic review and meta-analysis

Jongkonnee Thanasai [1], Chaimongkhon Chanthot [2], Anchalee Chittamma [3], Supphachoke Khemla [4], Atthaphong Phongphithakchai [5], Moragot Chatatikun [6,7], Jitbanjong Tangpong [6,7], Sa-ngob Laklaeng [6], Wiyada Kwanhian Klangbud [8,*]

[1] Faculty of Medicine, Mahasarakham University, Mahasarakham 44000, Thailand
[2] Project for the Establishment of the Faculty of Medicine, Nakhon Phanom University, Nakhon Phanom 48000, Thailand
[3] Department of Pathology, Faculty of Medicine Ramathibodi Hospital, Mahidol University, Bangkok 10400, Thailand
[4] Division of Infectious Diseases, Department of Internal Medicine, Nakhon Phanom Hospital, Nakhon Phanom 48000, Thailand
[5] Nephrology Unit, Division of Internal Medicine, Faculty of Medicine, Prince of Songkla University, Songkhla 90110, Thailand
[6] School of Allied Health Sciences, Walailak University, Nakhon Si Thammarat 80160, Thailand
[7] Research Excellence Center for Innovation and Health Products (RECIHP), Walailak University, Nakhon Si Thammarat 80160, Thailand
[8] Medical Technology Program, Faculty of Science, Nakhon Phanom University, Nakhon Phanom 48000, Thailand
[*]Corresponding author email: wiyadakwanhian@gmail.com

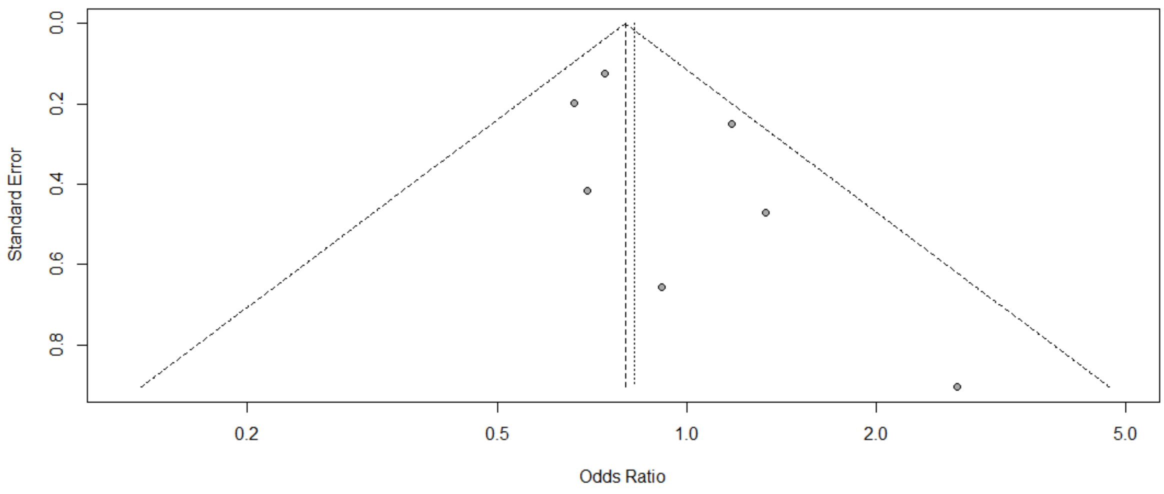

Supplementary Figure 1.1 Funnel of fatality

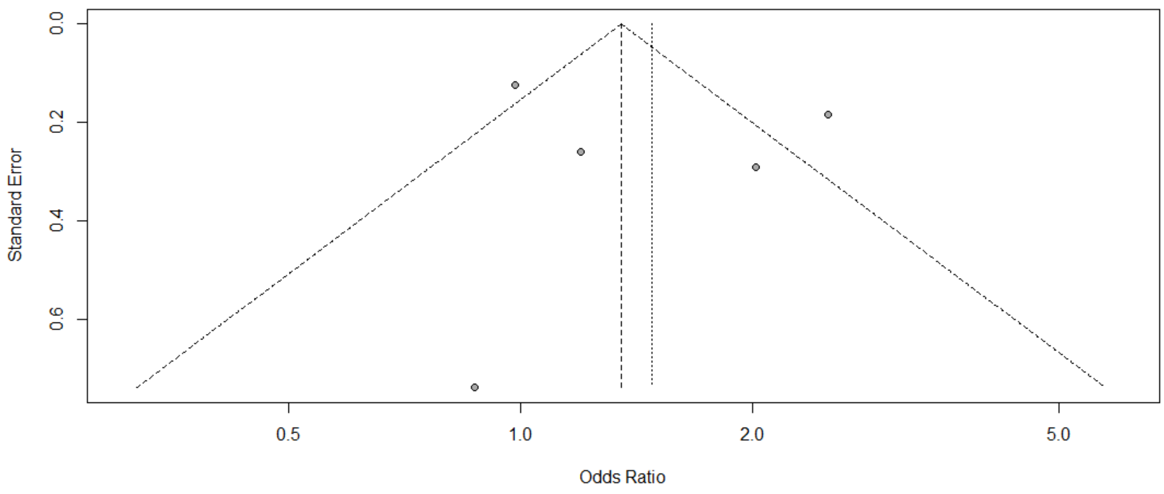

Supplementary Figure 1.2 Funnel of bacteremia

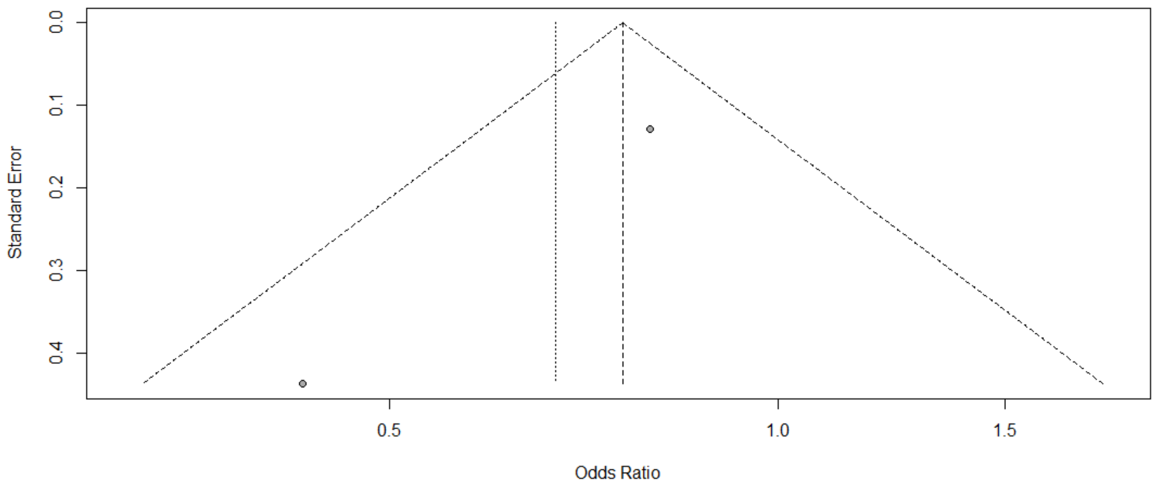

Supplementary Figure 1.3 Funnel of Septic shock

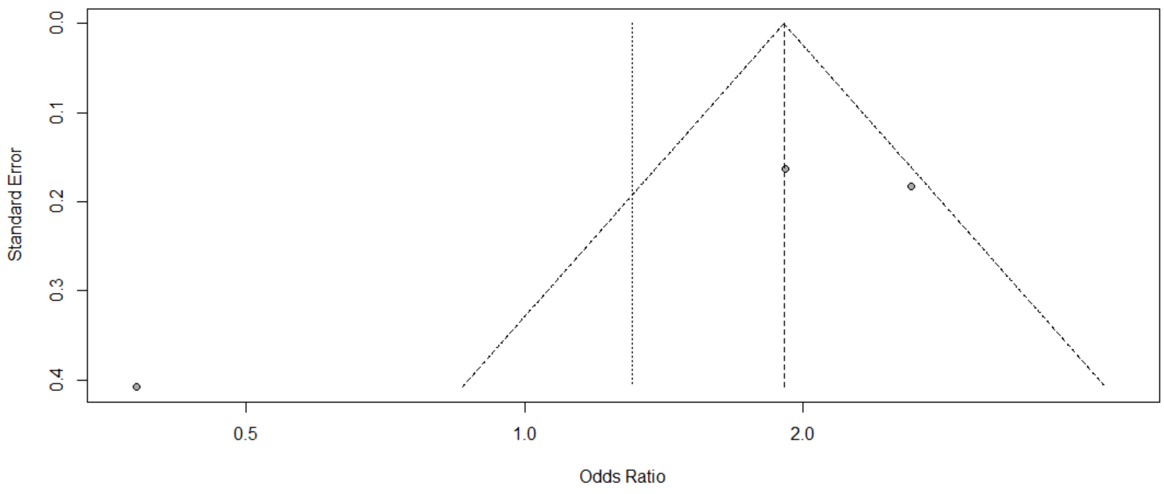

Supplementary Figure 1.4 Funnel of ICU admission
