# Supplementary material for: Diabetes Mellitus as a Risk Factor for Severe Disease and Mortality Among Patients with Melioidosis: A Systematic Review and Meta-Analysis
Source: Life (Basel). 2026 Feb 21;16(2):361. doi: 10.3390/life16020361 (PMC12942006; doi:10.3390/life16020361)
Supplement: Supplementary file 1 [file life-16-00361-s001.zip › Supplementary Table S1_Search stratergy.pdf]

Supplementary Table S1. Search strategy for database querying

PubMed

Search number	Query	Results
3	#1 AND #2	483
2	Melioidosis[Text Word] OR melioidosis[MeSH Terms] OR Burkholderia pseudomallei Infection[MeSH Terms] OR Burkholderia pseudomallei Infections[MeSH Terms] OR Infection, Burkholderia pseudomallei[MeSH Terms] OR Whitmore's Disease[MeSH Terms] OR Disease, Whitmore's[MeSH Terms] OR whitmore disease[MeSH Terms]	3,860
1	("diabetes mellitus"[MeSH Terms] OR Diabetes Mellitus[Text Word] OR DM[Text Word] OR diabet*[Text Word] OR diabetes[Text Word])	1,010,977

Embase

No.	Query	Results
#7	#4 AND #6	460
#6	#3 AND #5	1196576
#5	'diabetes'/exp OR 'diabetic'/exp OR 'diabets'/exp OR 'unspecified diabetes mellitus'/exp OR 'diabetes mellitus'/exp	1538998
#4	#1 OR #2	4633
#3	'diabetes mellitus':ti,ab,kw OR 'dm':ti,ab,kw OR 'diabetes':ti,ab,kw OR 'diabet*':ti,ab,kw	1501938
#2	melioidosis:ti,ab,kw	3985
#1	'b. pseudomallei infection'/exp OR 'burkholderia pseudomallei infection'/exp OR 'infection caused by burkholderia pseudomallei'/exp OR 'infection caused by pseudomonas pseudomallei'/exp OR 'infection due to burkholderia pseudomallei'/exp OR 'infection due to pseudomonas pseudomallei'/exp OR 'meliodosis'/exp OR 'p. pseudomallei infection'/exp OR 'pseudoglanders'/exp OR 'pseudomonas pseudomallei infection'/exp OR 'whitmore disease'/exp OR 'melioidosis'/exp	4055

Scopus

Search strategy	Result
(TITLE-ABS-KEY ("diabetes mellitus") OR TITLE-ABS-KEY (DM) OR (Diabet*) OR TITLE-ABS-KEY (Diabetes)) AND TITLE-ABS-KEY (Melioidosis) OR TITLE-ABS-KEY (Burkholderia pseudomallei infection)	1097
