# Supplementary material for: Diabetes Mellitus as a Risk Factor for Severe Disease and Mortality Among Patients with Melioidosis: A Systematic Review and Meta-Analysis
Source: Life (Basel). 2026 Feb 21;16(2):361. doi: 10.3390/life16020361 (PMC12942006; doi:10.3390/life16020361)
Supplement: Supplementary file 1 [file life-16-00361-s001.zip › Supplementary Table S2. Newcastle¿COttawa Scale (NOS) quality assessment.pdf]

Supplementary Table S2. Newcastle–Ottawa Scale (NOS) quality assessment of included studies

Study (Author, Year)	Study Design	Selection (★)	Comparability (★)	Outcome / Exposure (★)	Total Score (★)	Quality Rating
Birnie, 2019	Prospective cohort	★★★★	★★	★★★	★★★★★★★★☆ (8)	High
Corea, 2016	Case-finding study	★★★	★★	★★	★★★★★★☆☆☆ (7)	High
Currie, 2010	Prospective cohort	★★★★	★★	★★★	★★★★★★★★☆ (8)	High
Kronsteiner, 2019	Longitudinal study	★★★★	★★	★★	★★★★★★★☆☆ (8)	High
Kaewrakmuk, 2023	Retrospective cohort	★★★	★★	★★	★★★★★★☆☆☆ (7)	High
Prabhu, 2021	Retrospective cohort	★★★	★★	★★	★★★★★★☆☆☆ (7)	High
Smith, 2021	Retrospective analysis	★★★	★★	★★	★★★★★★☆☆☆ (7)	High
Stephens, 2016	Prospective database	★★★★	★★	★★	★★★★★★★☆☆ (8)	High
Stewart, 2017	Retrospective series	★★★	★★	★★	★★★★★★☆☆☆ (7)	High
Suputtamongkol, 1999	Case–control	★★★	★★	★★	★★★★★★☆☆☆ (7)	High
Tang, 2019	Retrospective study	★★★	★★	★★	★★★★★★☆☆☆ (7)	High
Wright, 2024	Prospective cohort	★★★★	★★	★★★	★★★★★★★★☆ (8)	High

Study quality was assessed using the Newcastle–Ottawa Scale (NOS) for observational studies. For cohort studies, the NOS evaluates three domains: **Selection** (maximum 4 stars), **Comparability** (maximum 2 stars), and **Outcome** (maximum 3 stars). For case–control studies, the Outcome domain is replaced by Exposure (maximum 3 stars).

Selection assesses representativeness of the exposed cohort/case definition, selection of the non-exposed/control group, and ascertainment of exposure. **Comparability** assesses adjustment for key confounders (at minimum age and sex, with additional adjustment where reported). **Outcome/Exposure** assesses outcome ascertainment, adequacy of follow-up, or exposure assessment, depending on study design.
